# Modeling refractive correction strategies in keratoconus

**DOI:** 10.1167/jov.21.10.18

**Published:** 2021-09-23

**Authors:** Jos J. Rozema, Gareth D. Hastings, Jason Marsack, Carina Koppen, Raymond A. Applegate

**Affiliations:** 1 Department of Ophthalmology, Antwerp University Hospital, Edegem, Belgium; 2 Department of Medicine and Health Sciences, Antwerp University, Wilrijk, Belgium; 3 College of Optometry, University of Houston, Houston, TX, USA; 4 Center for Innovation in Optics and Vision, School of Optometry, University of California, Berkeley, CA, USA

**Keywords:** keratoconus, refractive correction, statistical eye model, scleral lens, objective refraction

## Abstract

This work intends to determine the optimal refractive spectacle and scleral lens corrections for keratoconus patients using the visual Strehl (VSX) visual image quality metric and the SyntEyes models with the synthetic biometry of 20 normal eyes and 20 keratoconic eyes. These included the corneal tomography and intraocular biometry. A series of virtual spherocylindrical spectacle and scleral lens corrections spanning the entire phoropter range were separately applied to each eye, followed by ray tracing to determine the residual wavefront aberrations and identify the correction with the highest possible VSX (named a “focus”). To speed up calculations, a smart scanning algorithm was used, consisting of three consecutive scans over increasingly finer dioptric grids. In the dioptric space, the VSX pattern for normal eyes considered over the correction range for either spectacle or scleral lens corrections resembled an hourglass with one distinct focus and a quick drop in VSX away from that focus. For 18 of the 20 keratoconic eyes, the spectacle-corrected VSX pattern resembled a shell that in 9 of the 20 cases showed two foci separated by a large dioptric distance (13.3 ± 4.9 diopters). In keratoconic eyes, scleral lenses also produced hourglass patterns, but with a VSX lower than in normal eyes. The hourglass pattern in dioptric space shows how, in normal eyes, the refracting process automatically funnels practitioners toward the optimal correction. The shell patterns in keratoconus, however, present far more complexity and, possibly, multiple foci. Depending on the starting point, refracting procedures may lead to a local maximum rather than the optimal correction.

## Introduction

Keratoconus is characterized by progressive corneal thinning and irregularities in both the anterior and posterior corneal surfaces. These complex and progressive changes to corneal shape induce elevated levels of higher-order aberrations not correctable with spherocylindrical corrections. The disease typically becomes burdensome in the transitional life stage of early adulthood, when individuals are entering college, starting a family, beginning a career, and so on (Kymes et al., 2004).

In mild cases of the disease, toric spectacles can provide essentially normal visual acuity despite poorer visual quality (Zadnik, Barr, & Edrington, 1998; Wagner, Barr, Zadnik, & Collaborative Longitudinal Evaluation of Keratoconus (CLEK) Study Group, 2007). In moderate to advanced cases, rigid gas-permeable corneal lenses or scleral lenses provide better optical correction than spectacles because they more effectively decrease the refractive contribution of the anterior cornea and replace it with the comparatively smooth anterior contact lens surface. Nevertheless, it is good clinical practice to prescribe spectacles for when contact lenses are not being worn, highlighting the importance of identifying the best possible spectacle corrections.

Rigid gas-permeable lens corrections typically decrease total higher-order aberrations by about 60% (Choi, Wee, Lee, & Kim, 2007; Kosaki, Maeda, & Bessho, 2007; Hastings et al., 2019), but cannot mask the posterior corneal higher-order aberrations. In addition to decreasing aberrations, rigid gas-permeable lenses reverse the sign of key aberrations such as coma, causing visual distortions to smear in the opposite direction observed during spectacle or soft contact lens wear. This reversal is due to the corneal back surface (rather than the front surface) becoming the dominant origin of higher-order aberrations and light leaving the higher optical index of the cornea and entering the lower optical index of the aqueous humor (Chen & Yoon, 2008). As a result, finding the optimal refractive correction for keratoconus patients across these correction modalities is more challenging and variable than in typical eyes (Raasch et al., 2001). This challenge may be attributed to the commonly used subjective strategies to determine an optimal correction, which were developed to handle common cases of typical ametropia and astigmatism. But because keratoconic eyes have a wide range of refractive aberrations and are neurally adapted to their habitual blur (Hastings et al., 2021), it can be difficult even for the experienced practitioner to reach the best possible spherocylindrical correction for the patient.

To better understand the full complexity and limitations of correcting keratoconus with spherocylindrical corrections, we extended a model of uncorrected keratoconus (Rozema et al., 2017) to include standard spherocylindrical spectacles or contact lenses and calculated the resulting visual image quality. This refractive model was used to objectively scan through a large range of spherocylindrical combinations and evaluate plausible toric corrections for each specific eye to find local and global optima in visual image quality. These results are compared to similar models (Rozema, Rodriguez, Navarro, & Tassignon, 2016) for typical eyes, both with and without corneal astigmatism, to quantitatively and visually demonstrate the issues that the clinician and patient encounter. This includes demonstrating that the starting point of subjective refraction plays a much more important role in determining the final correction in keratoconus than in typical eyes. Ultimately, we hope that this type of analysis will lead clinicians to make more informed choices regarding spherocylindrical corrections for keratoconus patients that provide better visual image quality for each individual eye.

## Methods

### Correction model

The proposed correction model is an extension of the previously published SyntEyes (Rozema, Rodriguez, Navarro, & Tassignon, 2016) and SyntEyes KTC (Rozema et al., 2017) models. Unlike classic eye models that provide the biometry for a single idealized eye, the SyntEye models are stochastic computer programs that produce an unlimited number of plausible corneal and ocular biometry sets for both normal and keratoconic eyes. Because the parameters of these synthetic eyes have been proven statistically indistinguishable from real eyes for both models (Rozema et al., 2017; Rozema, Rodriguez, Navarro, & Tassignon, 2016), this process allows including a large physiological variation into the analysis. Hence, these models provide an attractive base from which to assess spherocylindrical refractive correction options in both normal eyes and those with keratoconus.

**Figure 1.**
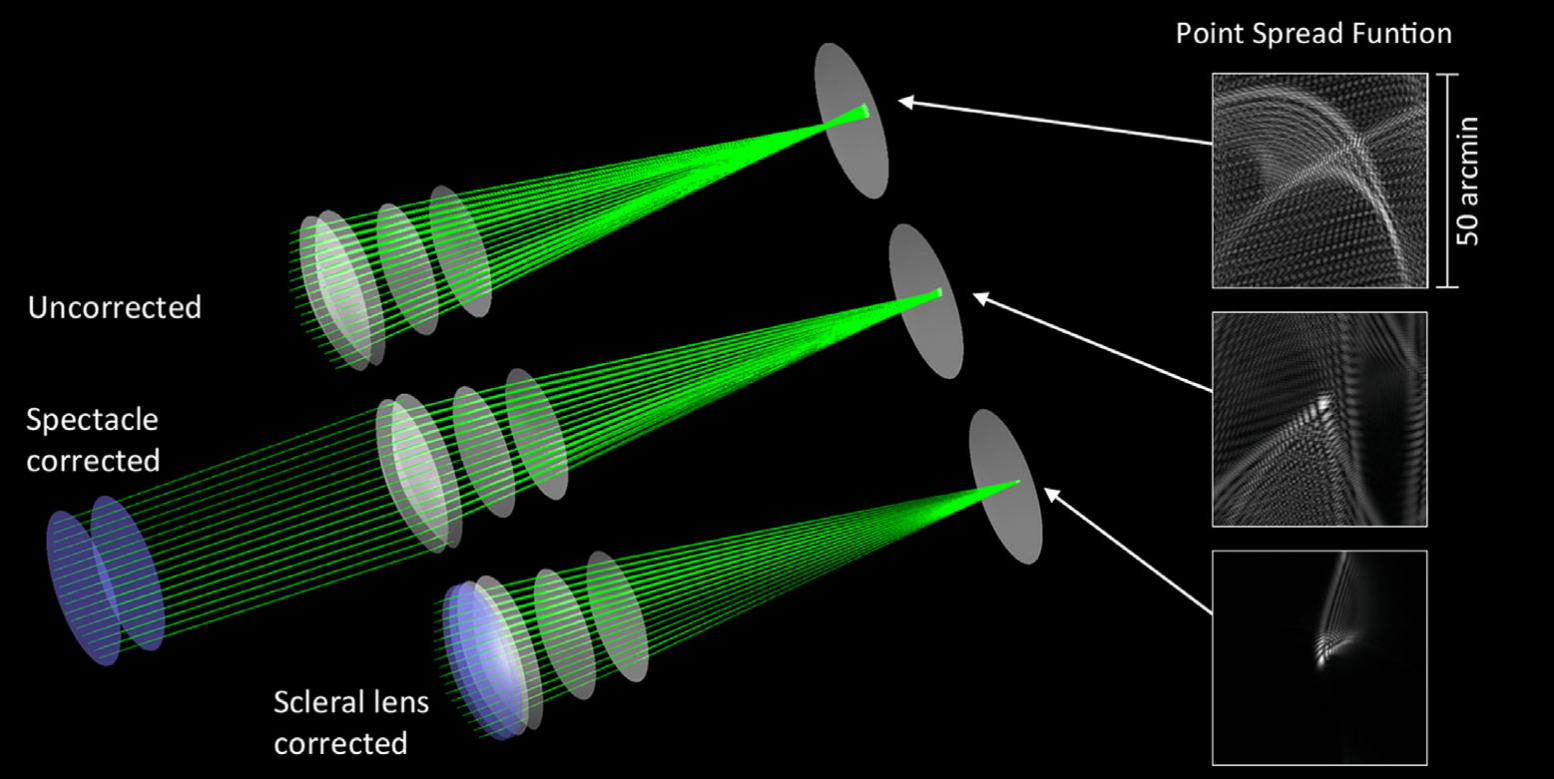
Optical models of an uncorrected keratoconic SyntEye and resulting point spread function (top), the same SyntEye with a spherical spectacle correction (middle), and with a spherical scleral lens correction (bottom). The point spread function tails were enhanced for visibility and are for illustrative purposes only rather than to calculate VSX.

The SyntEyes were generated and the corrections were implemented using custom ray tracing software (Matlab R2020a; Figure 1). The initial optical parameters of the spectacle and scleral lenses that were added to the existing models are listed in Table 1. The anterior curvature and asphericity of the correcting lenses were modified to simulate different correcting lens powers and orientations. Scleral lenses were simulated with a corneal vault of 0.325 mm and spectacles at a vertex distance of 12.000 mm. All corrections were centered along the optical axis of the ray tracing software. Ray tracing was performed through the correction and eye model using 121 rays over a 5-mm physical pupil diameter using a wavelength of 555 nm, fitted over the exit pupil of the lens-eye system with an eighth-order normalized Zernike series and scaled down to the physical pupil.

**Table 1.** Initial parameters of the refractive corrections.

	Radius of curvature, mm	Conic constant	Refractive index	Distance, mm
Spectacles				
Anterior surface	(Variable)	–(1/n_corr_) (Zadnik, Barr, & Edrington, 1998)	1.510	2.000
Posterior surface	100	–(n_corr_/n_air_) (Zadnik, Barr, & Edrington, 1998)	1.000	12.000
Scleral lenses				
Anterior surface	(Variable)	–(1/n_corr_) (Zadnik, Barr, & Edrington, 1998)	1.415	0.300
Posterior surface	7.20	–(n_corr_/n_tears_) (Zadnik, Barr, & Edrington, 1998)	1.3368 (Patel, Boyd, & Burns, 2000)	0.325

*n*_corr_ = refractive index of corrective lens; *n*_tears_ = refractive index of tear fluid; *n*_air_ = refractive index of air.

### Visual image quality assessment

Ray tracing of the generated SyntEyes provided the ocular wavefront errors, from which two estimates of the ocular refraction were calculated. The first, termed the Zernike refraction, used only Zernike terms of the second order, whereas the other, the Seidel refraction, used the terms from second to sixth orders (Thibos, Hong, Bradley, & Applegate, 2004). Finally, the visual Strehl (VSX) ratio (Marsack, Thibos, & Applegate, 2004; Thibos, Hong, Bradley, & Applegate, 2004) was calculated, a single-value visual image quality metric that combines the influence of optical aberrations with a measure of neural processing. This value represents a neural sharpness metric, calculated as the inner product of the point spread function with a neural weighting function, derived from photopic neural contrast sensitivity and normalized to the diffraction-limited case (Thibos, Hong, Bradley, & Applegate, 2004). Changes in the logarithm of VSX (logVSX) have been strongly correlated with changes in logarithm of the minimum angle of resolution visual acuity in eyes with keratoconus (Schoneveld, Pesudovs, & Coster, 2009; Ravikumar et al., 2013), and VSX has been used to successfully identify refractions that perform equally or better than subjective refraction in typical eyes (Hastings et al., 2017), as well as those of people with Down syndrome (Ravikumar, Benoit, Marsack, & Anderson, 2019).

### Optimized the scanning procedure

The modeling used a simulated through-focus method to identify optimal spherocylindrical corrections objectively. In a highly aberrated eye, there is often a wide range of spherocylindrical corrections in dioptric space that provide similar visual quality as reported by the patient. In some cases, there are even two regions of relatively good visual image quality (Marsack et al., 2014). Hence, we used an iterative process to search the dioptric space of a modern phoropter (Shumard et al., 2017). Because testing all possible corrections within this space (with the step sizes of 0.25 diopters [D], 0.25 D, and 2.5°, respectively), would lead to long calculation times, we used a smart scanning algorithm.

The smart scanning algorithm started by simulating each spherical correction in 0.25-D steps over a ±10 D range around the uncorrected Seidel refraction (spectacles) or a plano lens correction (scleral lens). The spherical correction with the highest logVSX value was then used as the starting point for three spherocylindrical scans within a range of ±7 D of the spherical range around this value for spectacles or ±3 D for scleral lenses. The first cylindrical scan was done in 1-D and 10° steps to separate the toric combinations that provide an improvement from those that either have no effect or worsen the visual image quality. Next, only those corrections with a VSX of greater than 0.03 were kept for the second cylindrical scan in which all corrections within ±0.5 D and ±5° of the remaining were calculated. Finally, a third scan was performed for all corrections within ±0.25 D and ±2.5° of points from the second scan with a VSX of greater than 0.01. The VSX cut-off of 0.01 was a trade-off between computation time and completeness of the VSX patterns presented in the Results.

The results are presented in the form of a double-angle three-dimensional point cloud in cylindrical coordinates, corresponding with the clinical sphere, cylinder, and axis (“correction space”). The value of logVSX is reflected in the color and size of the markers, with higher (better) values corresponding with cooler colors and larger markers. A local maximum region with highest VSX will be referred to as a best possible “focus” using spherocylindrical lenses. Areas were considered as distinct foci if they were separated by a VSX threshold of two-thirds times the maximal VSX value of the pattern.

## Results

The algorithms generated 20 normal (i.e., spherical refraction between ±10 D and no major astigmatism; Table 2) and 20 keratoconic SyntEyes for analysis. Maximal keratometry were 43.00 ± 1.00 D and 53.69 ± 4.85 D for normal and keratoconic eyes, respectively, and corresponding inferior–superior values (Rabinowitz & McDonnell, 1989) were 2.52 ± 1.00 D and –0.06 ± 0.18 D, respectively. Descriptive statistics of the results are given in Table 2. The topographies and correction space images of all SyntEyes are available in Supplement A.

**Table 2.** Descriptive statistics of SyntEyes used (mean ± standard deviation).

Model	Correction modality	Type	Sphere, D	Cylinder, D	*VSX*
Normal (*n* = 20)	Uncorrected (Seidel)	Residual	–0.74 ± 3.10	–0.78 ± 0.40	0.055 ± 0.091
	Uncorrected (Zernike)	Residual	–1.04 ± 3.00	–0.66 ± 0.35	0.055 ± 0.091
	Spherical spec corr.	Best corr.	–1.10 ± 2.37		0.158 ± 0.082
	Toric spec corr.	Best corr.	–0.38 ± 2.46	–0.73 ± 0.39	0.329 ± 0.040
	Spherical SCL corr.	Best corr.	–0.88 ± 2.15		0.214 ± 0.045
	Toric SCL corr.	Best corr.	–0.73 ± 2.26	–0.40 ± 0.13	0.387 ± 0.036
Keratoconus (*n* = 20)	Uncorrected (Seidel)	Residual	–2.84 ± 5.72	–4.27 ± 2.45	0.013 ± 0.011
	Uncorrected (Zernike)	Residual	–2.73 ± 3.56	–2.82 ± 1.77	0.013 ± 0.011
	Spherical spec corr.	Best corr.	–5.30 ± 4.68		0.034 ± 0.018
	Toric spec corr.	Best corr.	–4.31 ± 6.73	–6.53 ± 3.27	0.094 ± 0.028
	Spherical SCL corr.	Best corr.	–0.11 ± 2.06		0.198 ± 0.070
	Toric SCL corr.	Best corr.	+0.57 ± 2.18	–0.80 ± 0.58	0.312 ± 0.056

Corr = correction; SCL = scleral contact lens; Spec = spectacle.

### Normal eyes

The mean ± standard deviation uncorrected spherical refraction of the 20 normal eyes was –0.74 ± 3.10 D (range, –7.87 to +5.94 D) and the cylinder was –0.78 ± 0.40 D (range, –1.46 to –0.20 D). Increasing the complexity of correction modalities from uncorrected to toric scleral lenses progressively increased the VSX from 0.055 ± 0.091 (range, <0.0001 to 0.395) for uncorrected eyes to 0.387 ± 0.036 (range, 0.336, 0.473) for the best toric scleral lens correction (Table 2).

**Figure 2.**
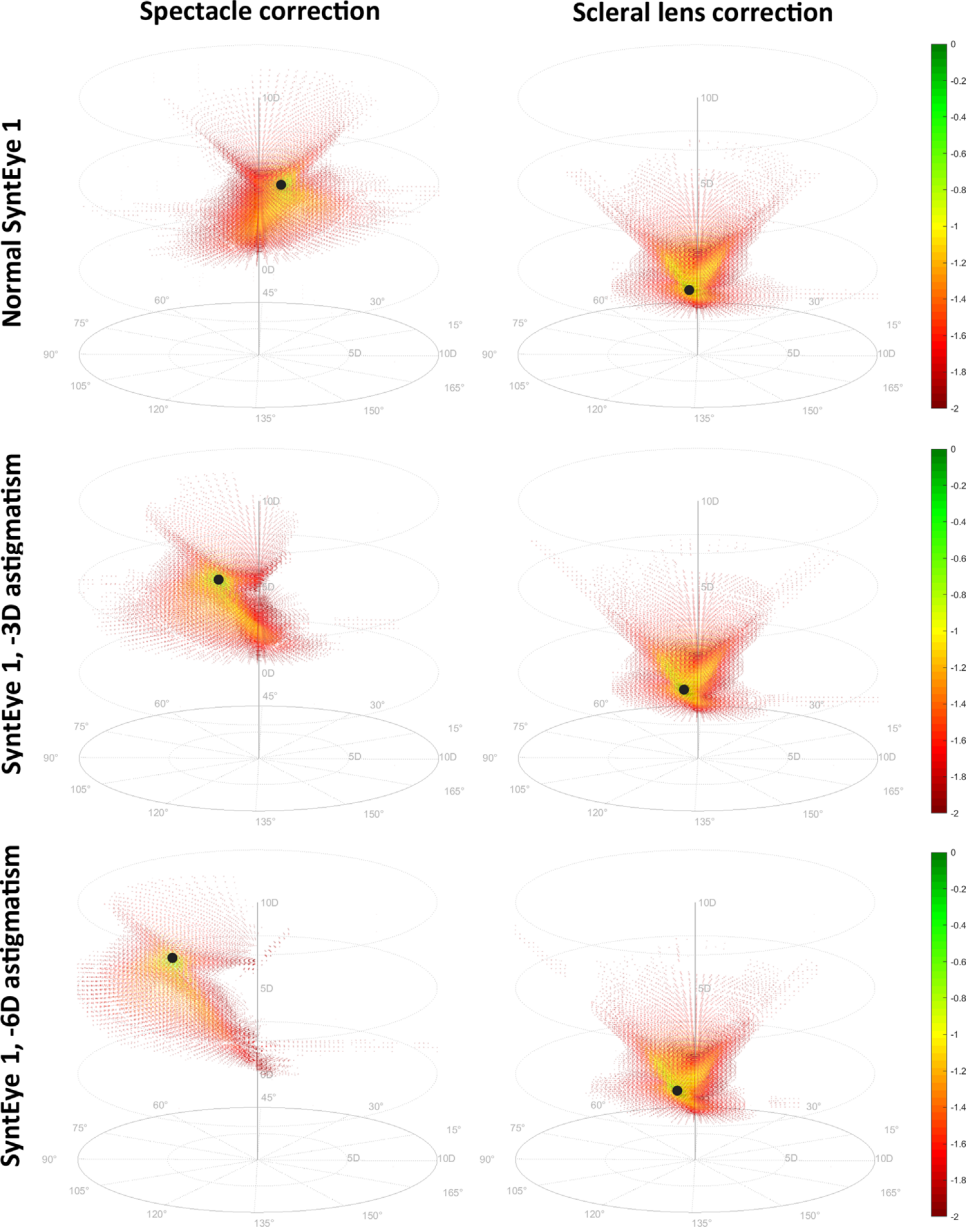
The log(VSX) in dioptric correction space for normal SyntEye 1 (top row), the same SyntEye with –3 D of astigmatism (center row) and with –6 D of astigmatism (bottom row). The black marker indicates the position of the focus (best correction).

Considering all spherocylindrical combinations in the correction space, the spectacle and scleral lens correction of all 20 normal SyntEyes showed a symmetric conical hourglass pattern in terms of VSX that is narrow near the optimal correction and broader as the spherical component moves away from this optimum in either positive or negative direction. The example in Figure 2 has a maximal VSX of 0.391.

To distinguish between normal corneal astigmatism and the complex irregularities of keratoconus, we added –3 D against-the-rule astigmatism to the anterior corneal surface. For spectacle corrections, this led to a similar conical pattern, albeit lopsided with a wider spherical range, a smaller cylindrical component, and a narrow spherical range for large cylinders. Here, the highest VSX was 0.175. When corneal astigmatism was increased to –6 D, the skewing of the hourglass pattern increased and the highest VSX decreased to 0.141. Using scleral lens corrections, the addition of corneal astigmatism to typical SyntEyes did not have a substantial effect on the pattern and would still produce compact clusters of good VSX located close to the central (spherical) axis (Figure 2). These patterns resemble the conical shape of the spectacle correction of the normal eye, albeit more compact and inverted with typically higher VSX values. All typical eyes had a single optimal refraction away from which visual image quality decreased monotonically.

### Keratoconus

The mean spherical refraction of the 20 uncorrected keratoconic eyes was –2.84 ± 5.72 D (range, –16.06 to +4.26 D) and the cylinder was –4.27 ± 2.45 D (range, –9.82 to –1.32D). Increasing the complexity of the correction modalities progressively increased VSX from 0.013 ± 0.011 (range, 0.0001–0.035) for uncorrected eyes to 0.312 ± 0.056 (range, 0.192–0.397) for the best toric scleral lens correction (Table 2).

**Figure 3.**
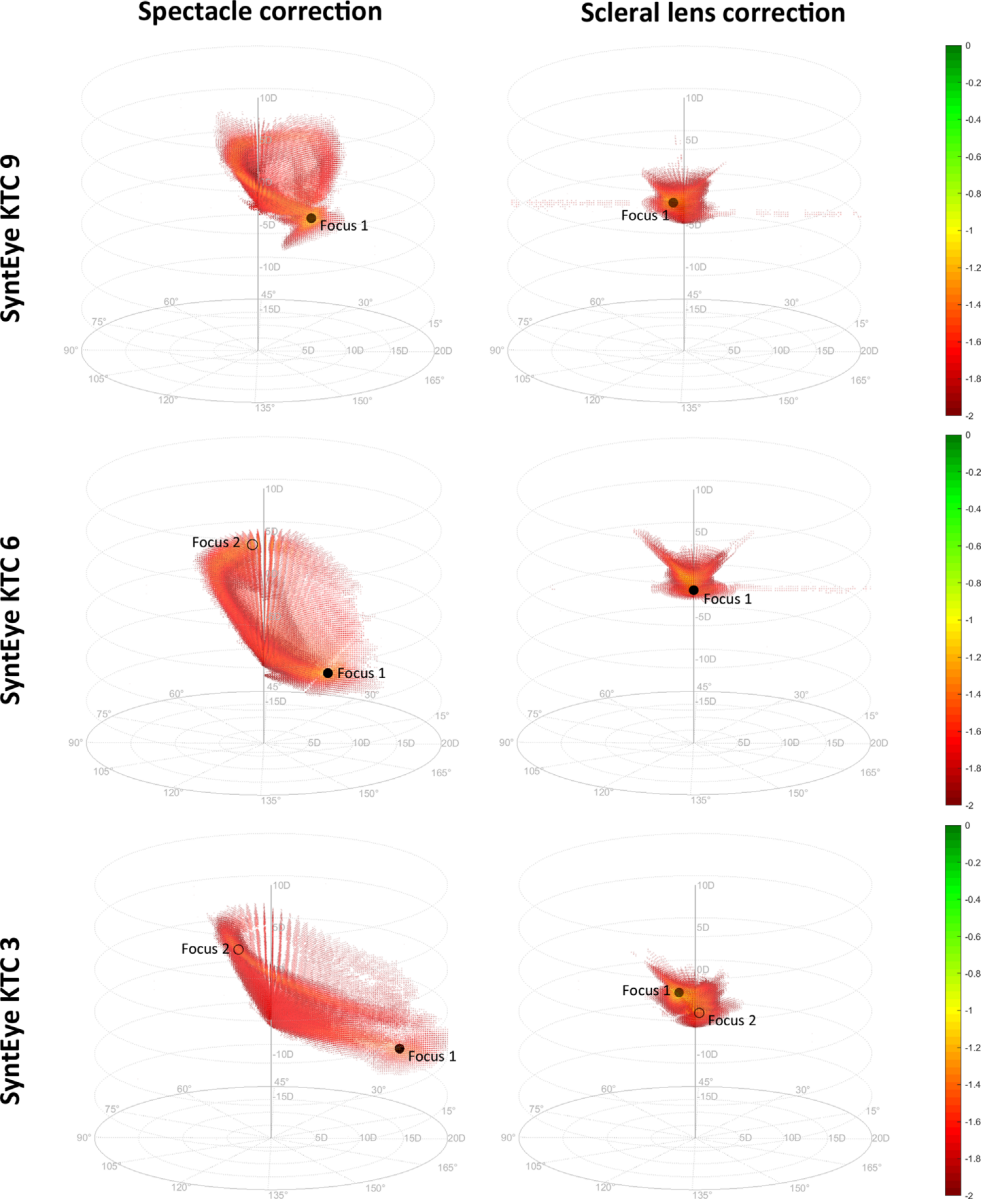
The log(VSX) in correction space for three examples of keratoconic SyntEyes: one with a single optimum for spectacle corrections (top), one with two foci for spectacles and one for scleral lenses (center), and one with two foci for spectacles and two for scleral lenses (bottom). Black markers indicate the foci; solid marker represents the focus with highest VSX.

Spectacle corrections in keratoconus generally lead to shell-like patterns of good VSX in correction space that had either one or two foci (Figure 3). This shell-like pattern, sometimes with branches, was seen in 18 of 20 keratoconus eyes and was more pronounced in severe cases. The other 2 of these 18 eyes had a pattern resembling a loose knot in which the “shell” was not developed fully. Both of these cases were a relatively early stage of keratoconus (see Supplement A). Figure 3 shows several examples of these shell patterns, the first of which showed one focus in both spectacle and contact lens corrections. The second example had two foci with relatively low VSX values (0.071 and 0.060, respectively), but were dioptrically very far apart (centered at –11.5 D –7.25 D × 177.5° and +1 D –8.0 × 50°, respectively). The scleral lens corrections of these two SyntEyes formed a more compact pattern, as typically seen in the normal eyes. The third example is an advanced case with two foci for the spectacle correction (centered at –10 D –15 D × 7.5° with VSX = 0.055 and +3.25 D –4.75 × 110° with VSX = 0.042, respectively), but also for the scleral lens correction (centered at +1.5 D –2 D × 102.5° with VSX = 0.247 and –1.25 D –0.5 × 17.5° with VSX = 0.180, respectively).

Overall, 9 of 20 keratoconus eyes had two foci for spectacle corrections (i.e., with local maxima separated by values of <2/3·max[VSX]) and 5 of 20 for scleral lens correction. On average, the difference in cylinder axes of these foci were 96.4 ± 32.1° for spectacles and 65.6 ± 20.5° for scleral lenses. Meanwhile, the dioptric distances between the foci were 13.3 ± 4.9 D and 1.7 ± 0.7 D, respectively.

**Figure 4.**
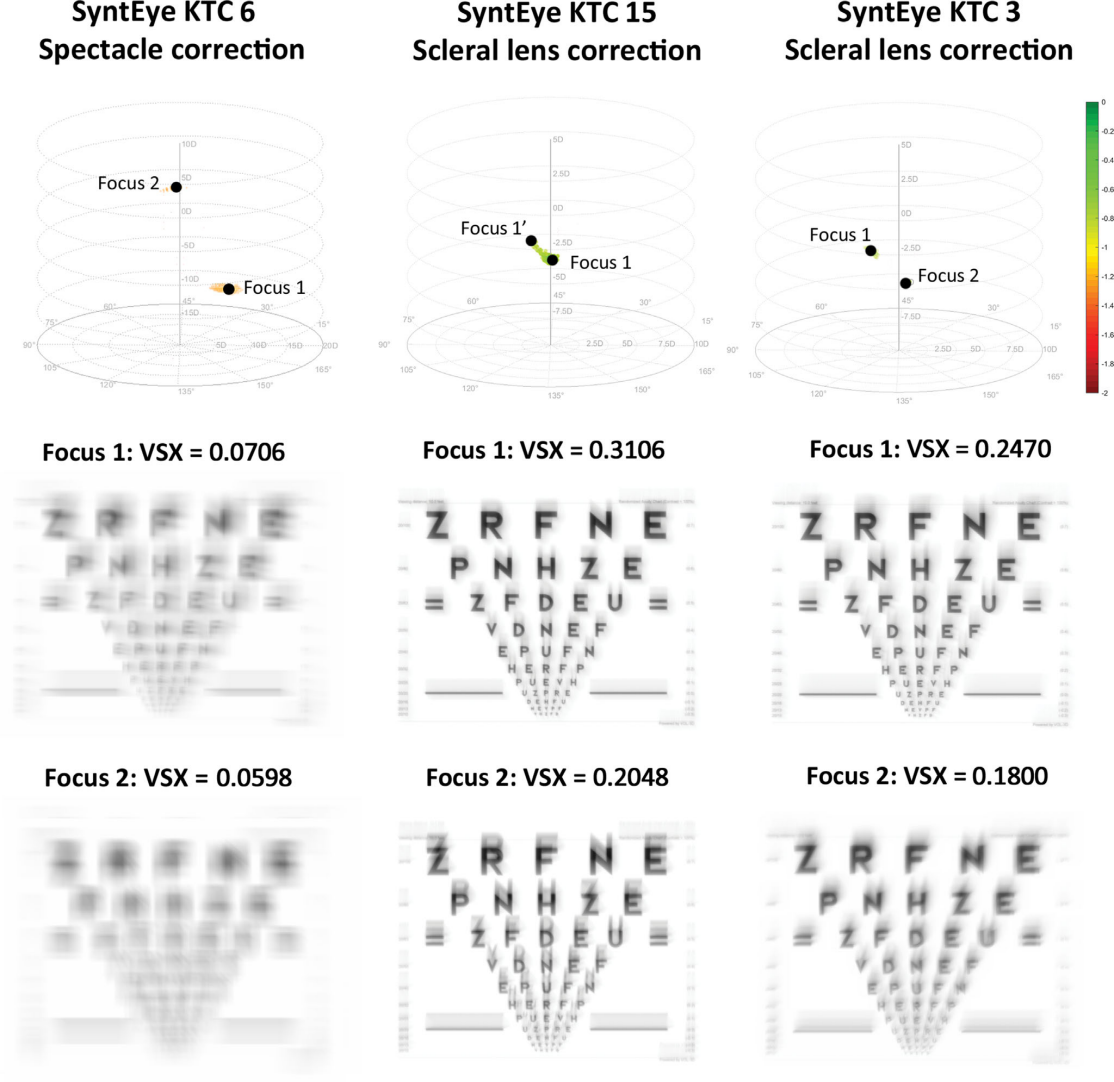
Simulated logarithm of the minimum angle of resolution charts illustrating the visual quality in the two foci in correction space for the spectacle correction of SyntEye KTC 15 (first column), the scleral lens correction of SyntEye KTC 15 (second column), and the scleral lens correction of SyntEye KTC 3 (third column).

Once the corrections with the highest VSX are known, one can simulate the retinal image of a visual acuity letter chart for a SyntEye wearing these corrections (Figure 4). For example, the two foci of keratoconic SyntEye 6 with spectacle correction shows a rather large difference in visual image quality. SyntEye 15 with scleral lens correction, in contrast, has a single extended focal region of variable VSX. Finally, keratoconic SyntEye 3 with scleral lens correction also shows a clear difference between both foci.

## Discussion

This work explored a number of aspects of correcting eyes with regular and irregular corneas to assess why, even with scleral lenses, it can be difficult to achieve good visual quality in eyes with keratoconus. Before these interpretations are elaborated upon, we first show that the modeling of synthesized eyes is representative of real eyes.

### Comparisons with the literature

Typical spectacle-corrected SyntEyes were included as a control for the modeling. Spherocylindrically best-corrected VSX values (0.329 ± 0.040) of the SyntEyes were similar to the values of 36 real myopic eyes objectively corrected with spectacles (VSX 0.334 ± 0.092) (Hastings et al., 2017). We are unaware of any literature that has reported visual image quality values of typical eyes wearing scleral lenses; however, the synthesized eyes modeled here (0.387 ± 0.036) were within the pupil size- and age-matched 95% VSX norms of spherocylindrically objectively best-corrected typical eyes (Hastings, Marsack, Thibos, & Applegate, 2018). Overall, the model's agreement with typical eyes indicated that the simulated corrections were modeled effectively, despite simplifications and assumptions regarding the form of the spectacle lenses.

One purpose of this work was to assess the challenging process of refracting eyes with keratoconus. Thus, the modeling is compared with independently collected data (Shumard et al., 2017; Hastings et al., 2019) not used in the development of the models. The VSX of the keratoconic SyntEyes with toric spectacle correction (0.094 ± 0.028) and spherical lens corrections (0.198 ± 0.070) agreed with corresponding values of real keratoconic eyes (0.118 ± 0.141 and 0.190 ± 0.086, respectively) (Shumard et al., 2017; Hastings et al., 2019). Note that these values remain far below those of normal eyes with spectacle corrections (0.329 ± 0.040).

### Refraction in keratoconus

Having shown that the modeling was representative of real eyes, it can be used to investigate the question of why increased higher-order aberrations make refracting difficult in keratoconus. As mentioned in the Introduction, there is an inherent challenge in trying to compensate for elevated higher-order aberrations with a lower-order correction. This process is made more difficult because, unlike normal eyes, eyes with keratoconus do not necessarily have one unique area of optimal dioptric correction (Figure 4) (Marsack et al., 2014) and visual image quality used by patients to guide the subjective refraction is poorer in keratoconus. Because subjectively refracting an eye is a sequential process in which the practitioner seeks the optimal correction based on the starting point, as well as the binary better or worse responses by the patient, this modeling illustrates how the process can direct the practitioner to a local, rather than the global, maximum. This is seen in both spectacle and scleral lens corrections (in 10 of 20 and 5 of 20 SyntEyes, respectively), with a considerably larger dioptric distances for the spectacles. Consequently, depending on their starting point (e.g., Seidel or Zernike refraction, autorefraction, retinoscopy, or habitual correction), practitioners can easily reach a local maximum focus rather than the globally optimal correction. In many cases, it is plausible that the global maximum may never be reached because one must traverse a dioptric space with worsening visual image quality between the foci before it improves as one approaches the global maximum (Figure 3). Moreover, it is important to remember that, although the best possible spectacle corrections are often inferior to the visual quality that can be accomplished with scleral lenses, patients cannot continuously wear scleral lenses and will need spectacles for the times between lens wear.

In essence, the patterns in the scatterplots are a series of through-focus spherical scans in the presence of a cylindrical correction oriented along a certain axis. As such, any vertical line through these patterns likely represents something similar to the interval of Sturm, the distance between the horizontal and vertical foci of an astigmatic eyes. This finding is supported by the observations that keratoconic SyntEyes with two regions of highest VSX find those regions on opposite sides of the pattern, both in the vertical direction (sphere) and in the horizontal direction (cylinder and orientation). This reasoning is not the entire explanation, however, as in typical eyes with a regular corneal astigmatism there was only one compact region of highest VSX. Meanwhile, the shell-like shape is probably related to how the different regions of the keratoconic cornea sequentially gain dominance in the through-focus scans using only a spherocylinder correction. As the spherical power of the correction moves from more positive to more negative, the position where a given ray is effectively incident on the cornea changes. One can see how the lower part of the pattern would correspond with the negative spherical corrections required to correct the high power of the steep cone area, whereas positive or low-negative corrections would correct the flatter superior cornea. The scleral lens corrections, in contrast, lead to highly compact patterns, but the focus can be extended under the influence of the residual corneal astigmatism (see Supplement A). In this sense, VSX is a useful visual image quality metric for the current purpose as it uses all visually relevant information of the light passing through the limiting aperture of the eye. Using VSX is also advantageous in rotationally asymmetric cases, such as keratoconus, because one can be more confident about the correct centration and application of neural weighting functions in the spatial domain (VSX) rather than in the Fourier domain. Nevertheless, changes in pupil size might affect the appropriateness of the objectively optimal correction, which is a topic of ongoing investigation.

Note that these results also have several limitations toward translation into clinical applications. The first is the use of VSX to assess the visual image quality. On its own VSX does not predict absolute visual acuity, but changes in its logarithm (logVSX) are strongly correlated with changes in logarithm of the minimum angle of resolution visual acuity (Schoneveld, Pesudovs, & Coster, 2009; Ravikumar et al., 2013). As such, the patterns in Figures 2, 3, and 4 should be considered as qualitative indications of where in correction space the best foci of those eyes would be under a spherocylindrical correction, rather than actual visual acuity that these eyes might be able to reach. VSX is able to do this in a very robust way, but is conceivable that better, more suitable visual image quality metrics will be developed that outperform VSX in the future. Further, although the upper bound of VSX for spherocylindrical correction in normal eyes has been defined (Hastings, Marsack, Thibos, & Applegate, 2018), the lower bound where VSX is meaningful has not. This finding is relevant to many of the VSX values reported here for the keratoconic eyes.

Next, the analysis used SyntEyes rather than real eyes. Although the results were in agreement with real eyes and the methods described could be applied to real eye data, the challenge is that clinical data are too often incomplete to make a reliable personalized whole-eye model because parts of the lens biometry are missing. SyntEyes solve this issue by making several assumptions that work on the population level, but might lead to errors if used to estimate the lens biometry of an individual eye (Rozema, Rodriguez, Navarro, & Tassignon, 2016) that could lead to incorrect estimates of the lenticular wavefront. Given that the wavefront aberrations of the crystalline lens interact with those of the cornea to minimize the total wavefront (Artal, Berrio, Guirao, & Piers, 2002), the known lenticular aberrations of the SyntEyes may be beneficial for this current proof-of-concept analysis. In a clinical setting, and in the absence of a clinical way to reliably estimate of the crystalline lens aberrations (Atchison et al., 2016), one might use a crystalline lens power calculation instead, if necessary, in an iterative algorithm. Another limitation of the current model is that the corrective lenses are assumed to be stable, on-axis and with a generic vault, thickness, and form. Moreover, the pupil size of 5 mm used here is rather large, which might have reduced VSX. The influence of these variables is part of currently ongoing studies. Finally, the calculation times are too long to be used in daily practice (3–8 hours per eye), even when using parallel computing on a high-end computer, which might become a moot point as computational processing technology advances.

## Conclusion

The main source of difficulties when prescribing spectacles for a patient with keratoconus lies in the complex variations of the visual image quality within dioptric correction space that may give rise to separate, dioptrically distant foci representing the best possible spectacle corrections for that eye. This complexity means that practitioners may not always be able to reach these foci through the normal refracting process, ultimately leading to a suboptimal spectacle correction for the patient.

## Supplementary Material

Supplement 1
