# Supplementary material for: Modeling refractive correction strategies in keratoconus
Source: J Vis. 2021 Sep 23;21(10):18. doi: 10.1167/jov.21.10.18 (PMC8475278; doi:10.1167/jov.21.10.18)

Visual Strehl patterns of

20 Normal SyntEyes

(Cutoff 0.01)

SyntEye 1 Spectacle correction

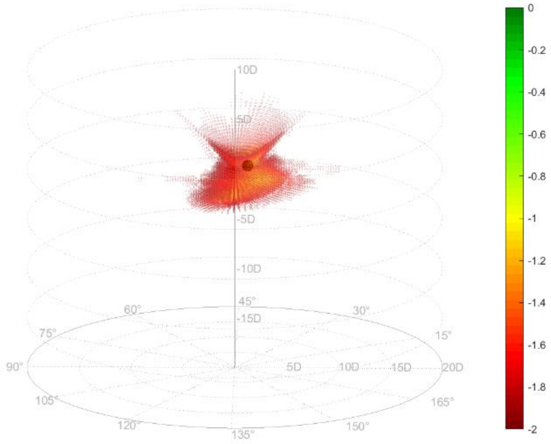

SyntEye 1 Scleral lens correction

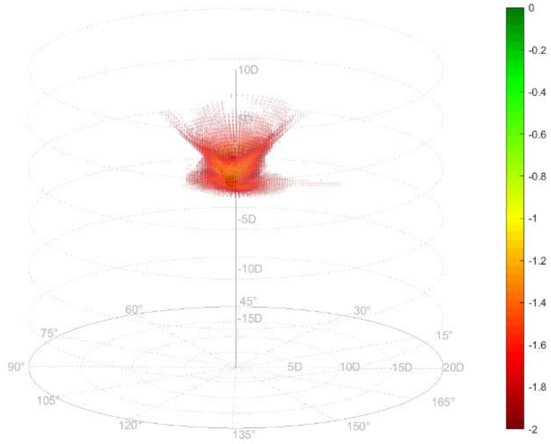

SyntEye 2 Spectacle correction

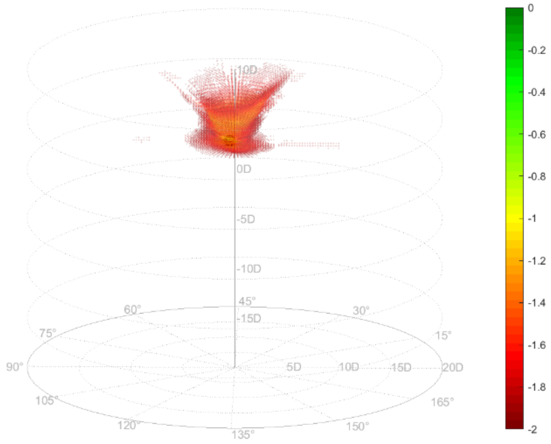

SyntEye 2 Scleral lens correction

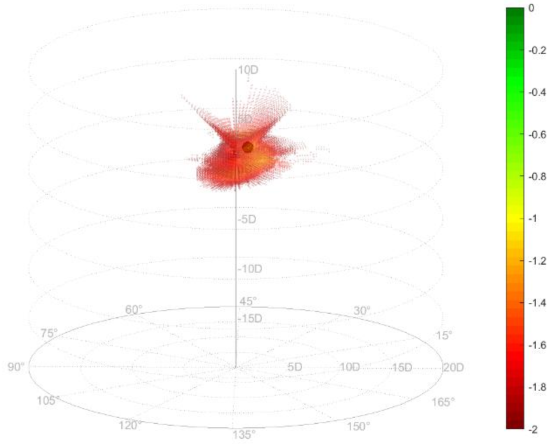

SyntEye 3 Spectacle correction

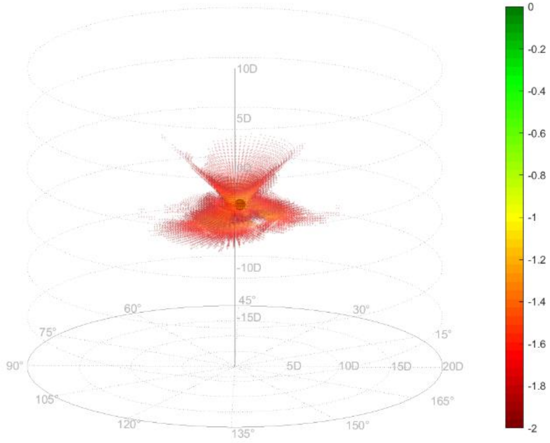

SyntEye 3 Scleral lens correction

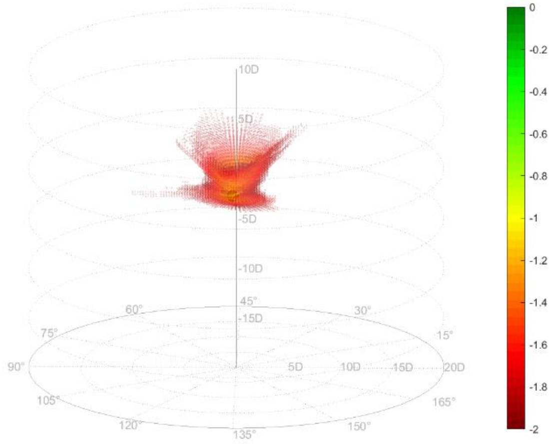

SyntEye 4 Spectacle correction

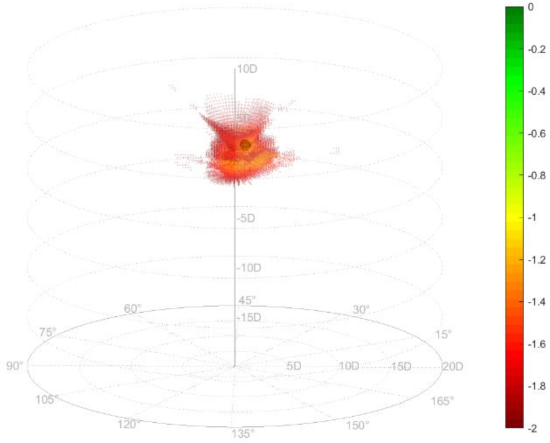

SyntEye 4 Scleral lens correction

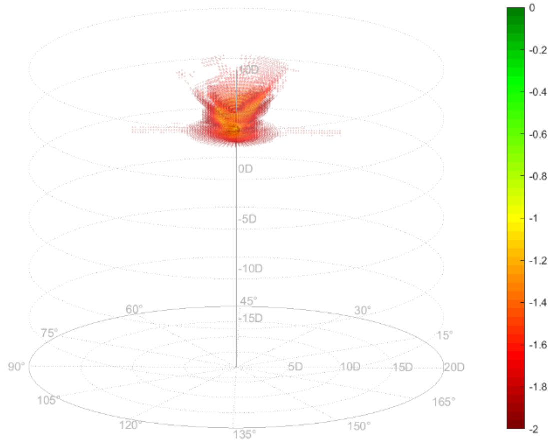

SyntEye 5 Spectacle correction

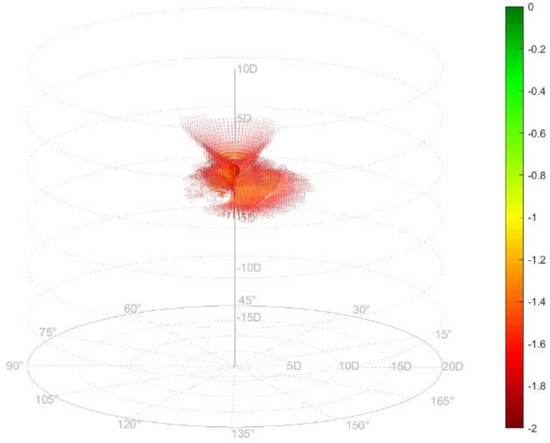

SyntEye 5 Scleral lens correction

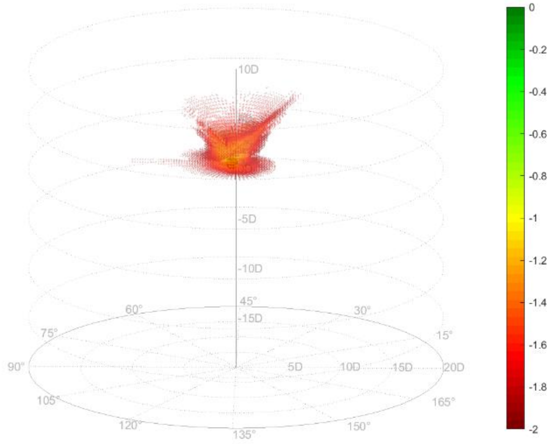

SyntEye 6 Spectacle correction

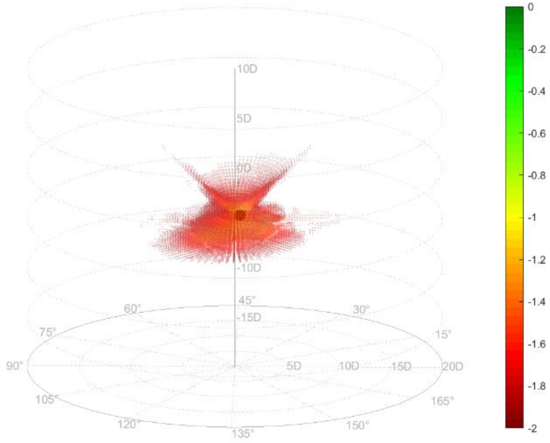

SyntEye 6 Scleral lens correction

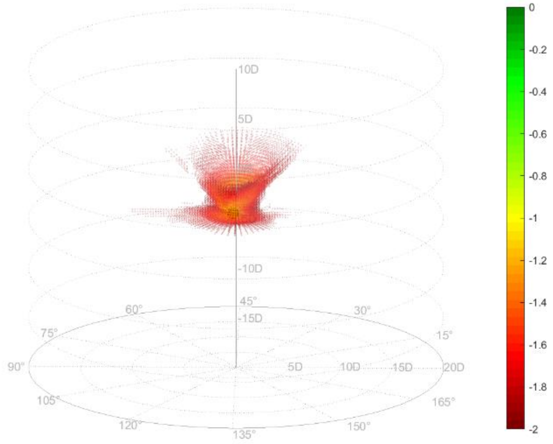

SyntEye 7 Spectacle correction

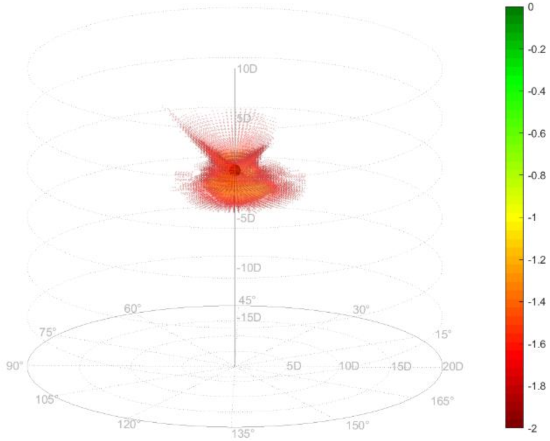

SyntEye 7 Scleral lens correction

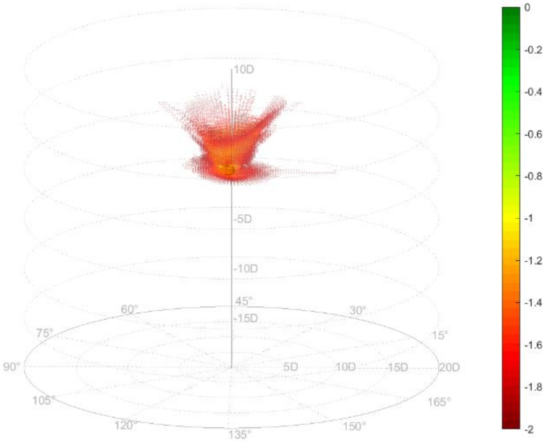

SyntEye 8 Spectacle correction

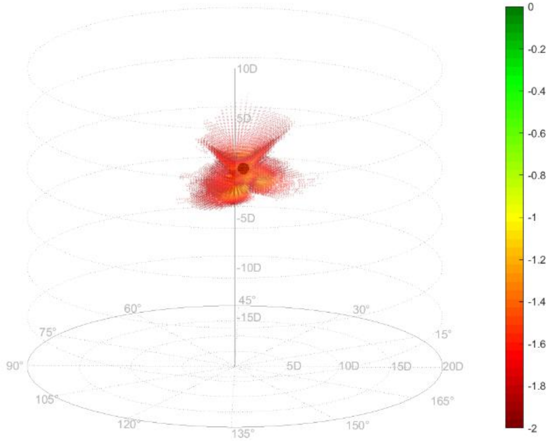

SyntEye 8 Scleral lens correction

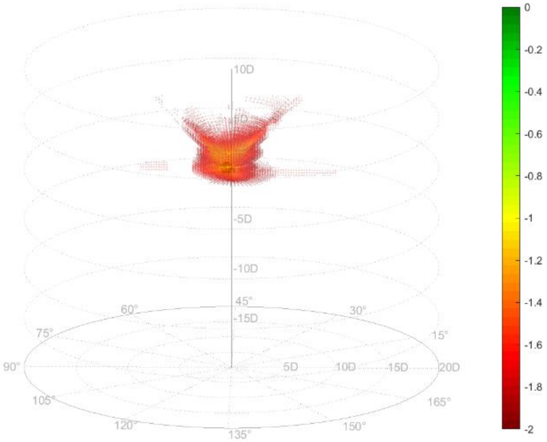

SyntEye 9 Spectacle correction

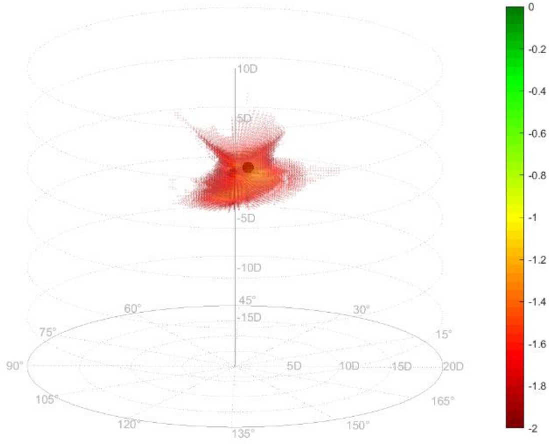

SyntEye 9 Scleral lens correction

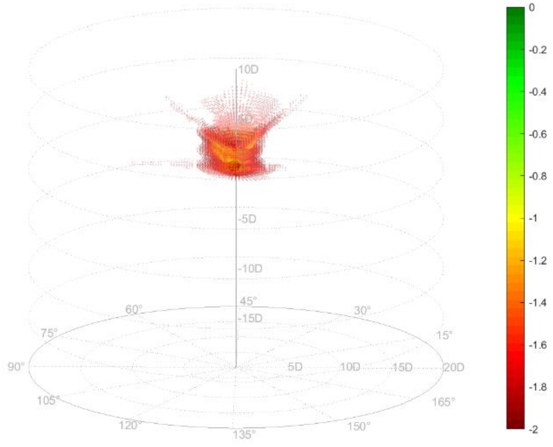

SyntEye 10 Spectacle correction

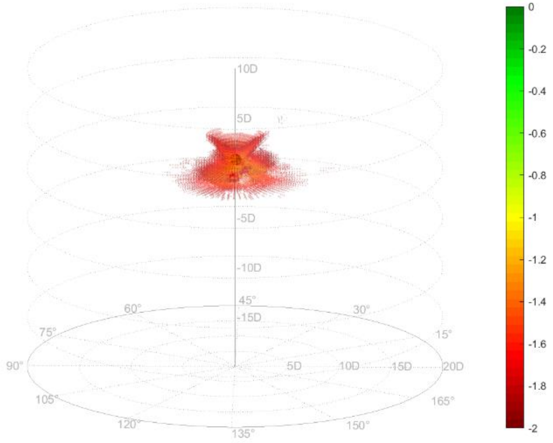

SyntEye 10 Scleral lens correction

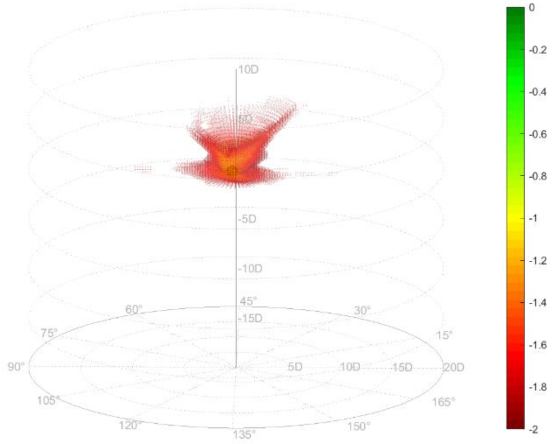

SyntEye 11 Spectacle correction

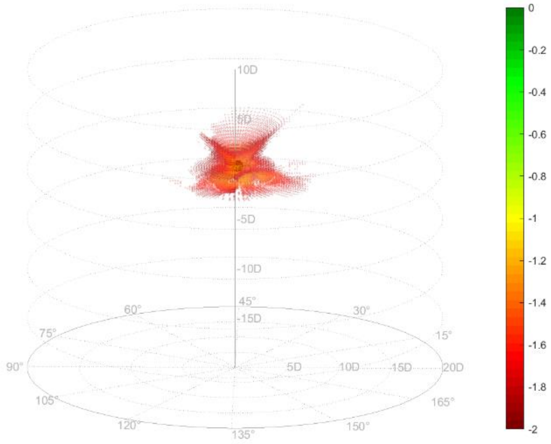

SyntEye 11 Scleral lens correction

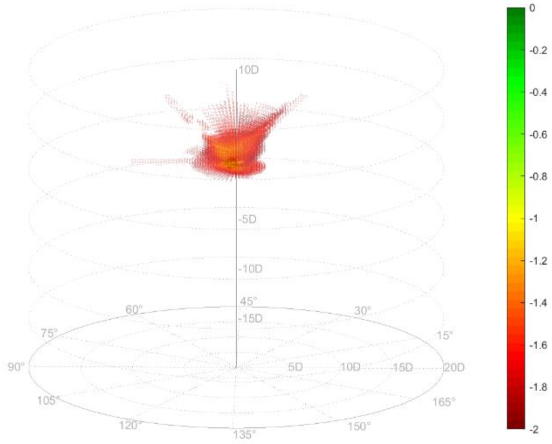

SyntEye 12 Spectacle correction

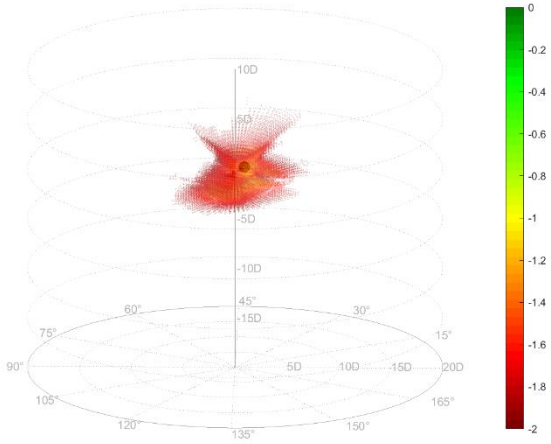

SyntEye 12 Scleral lens correction

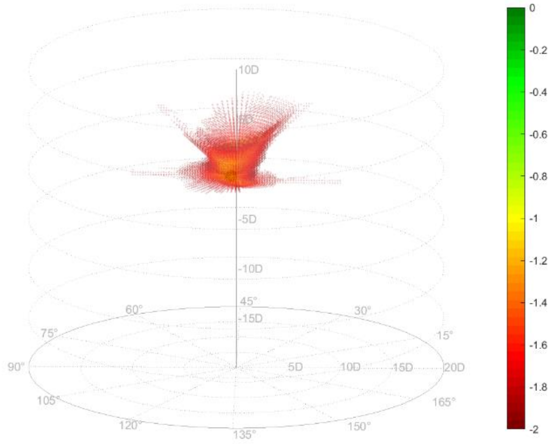

SyntEye 13 Spectacle correction

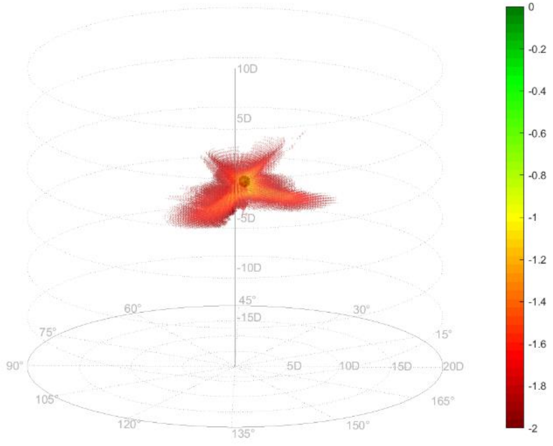

SyntEye 13 Scleral lens correction

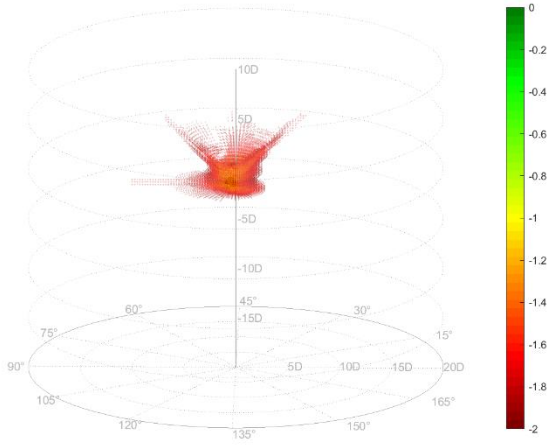

SyntEye 14 Spectacle correction

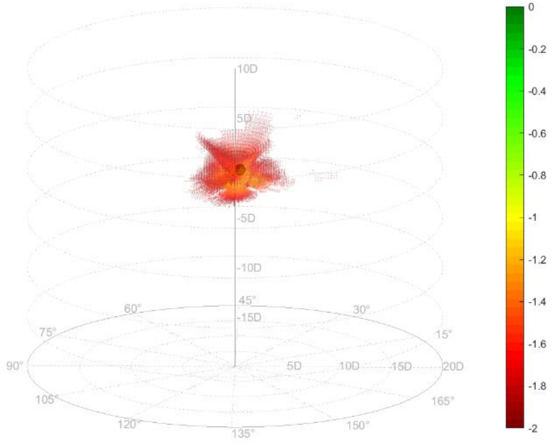

SyntEye 14 Scleral lens correction

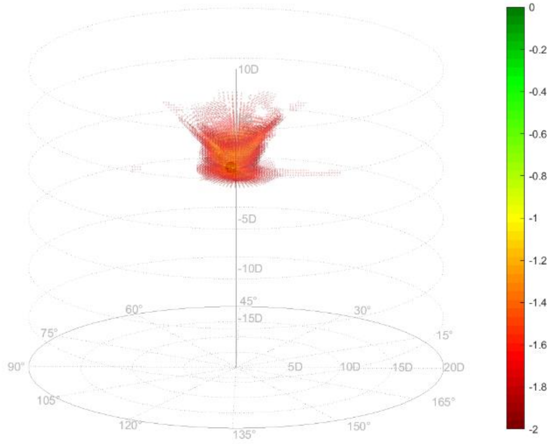

SyntEye 15 Spectacle correction

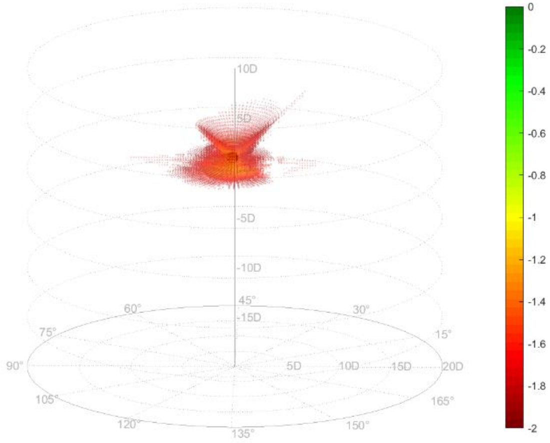

SyntEye 15 Scleral lens correction

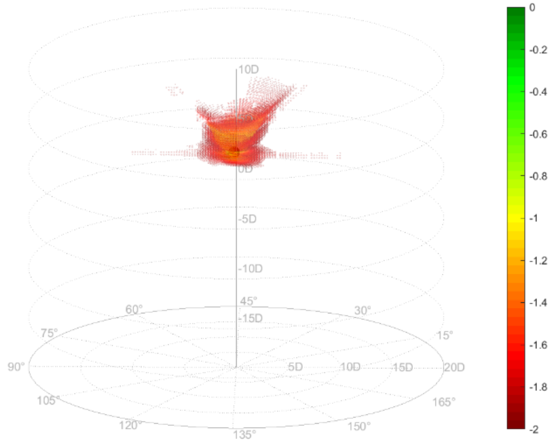

SyntEye 16 Spectacle correction

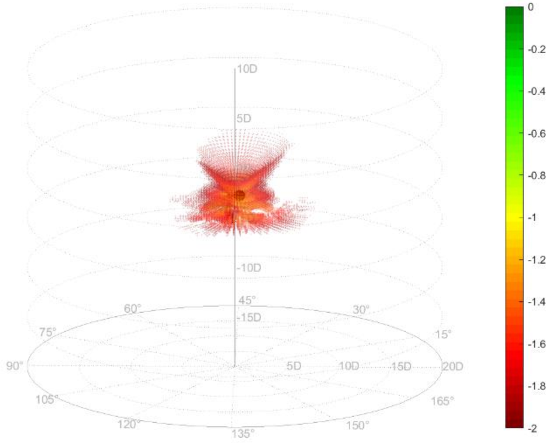

SyntEye 16 Scleral lens correction

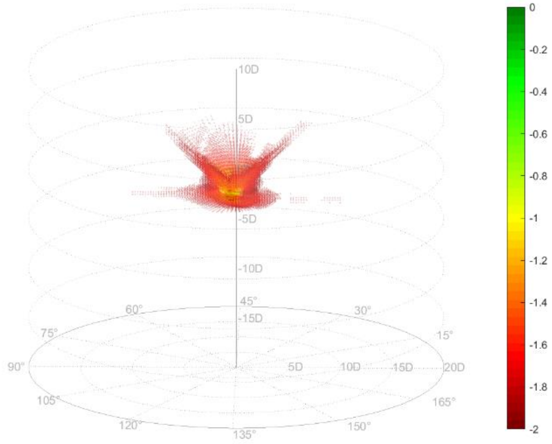

SyntEye 18 Spectacle correction

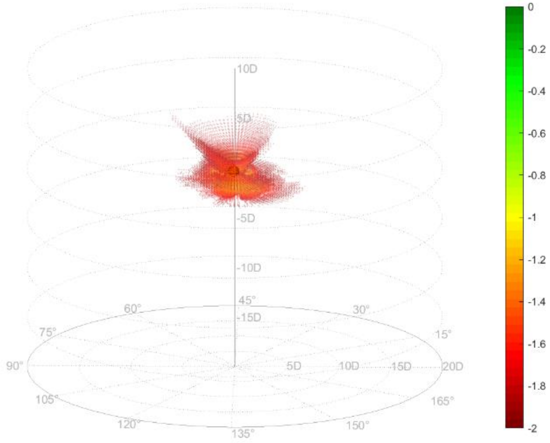

SyntEye 18 Scleral lens correction

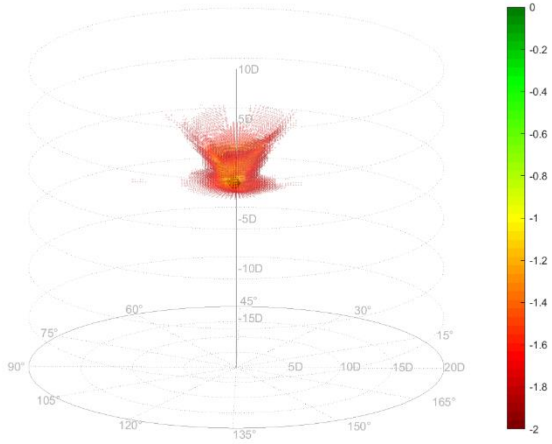

SyntEye 20 Spectacle correction

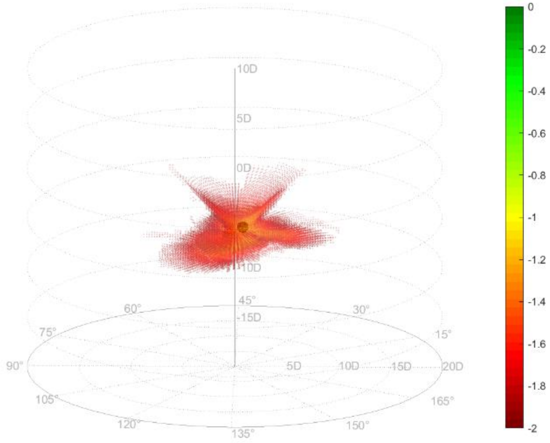

SyntEye 20 Scleral lens correction

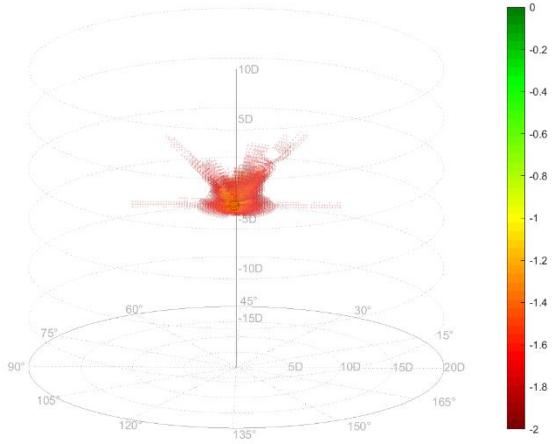

SyntEye 21 Spectacle correction

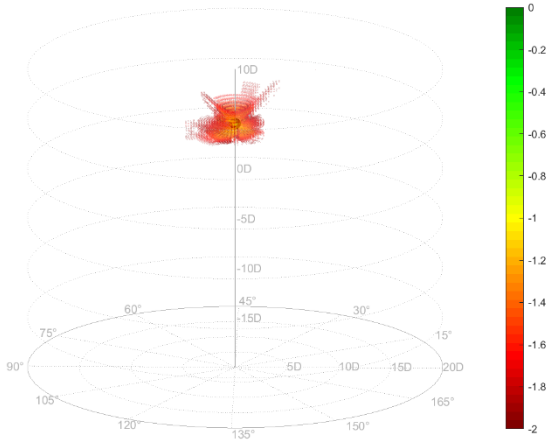

SyntEye 21 Scleral lens correction

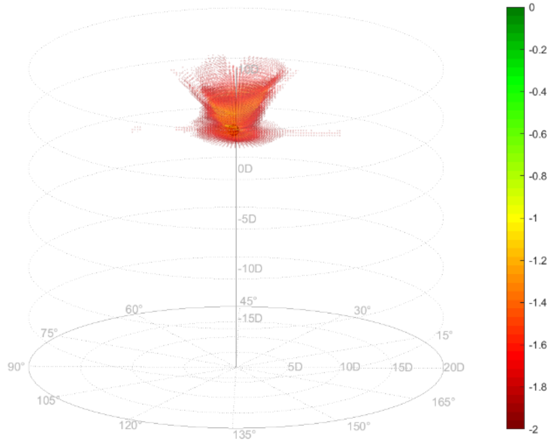

SyntEye 23 Spectacle correction

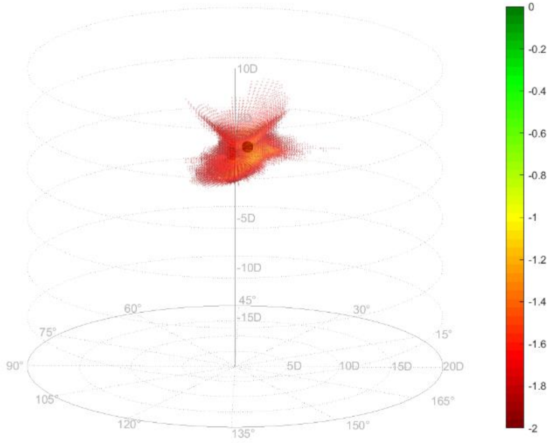

SyntEye 23 Scleral lens correction

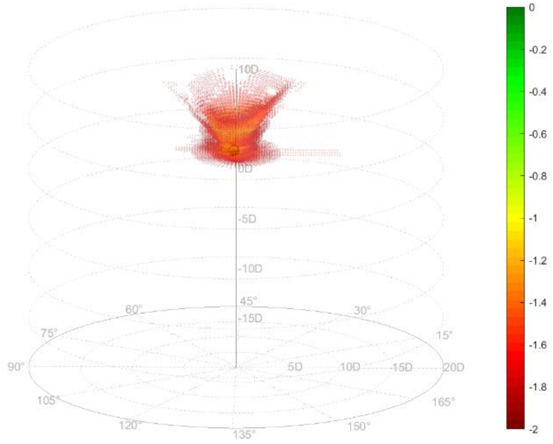

Visual Strehl patterns of

20 Keratoconic SyntEyes

(Cutoff 0.01)

SyntEye KTC 1 Spectacle correction

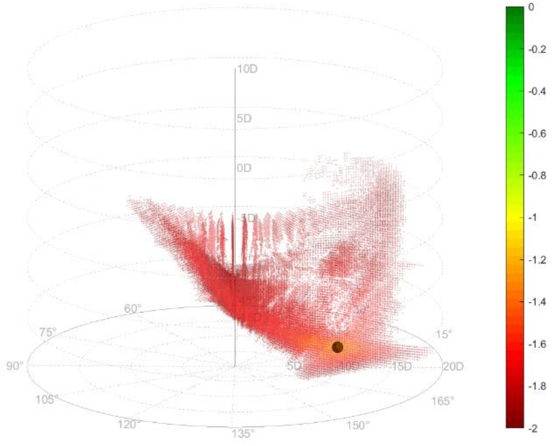

SyntEye KTC 1 Scleral lens correction

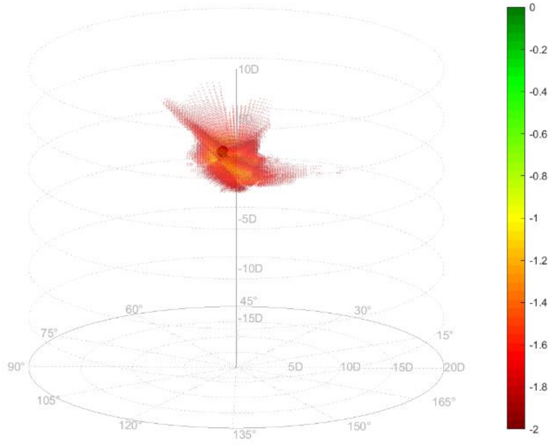

SyntEye KTC 2 Spectacle correction

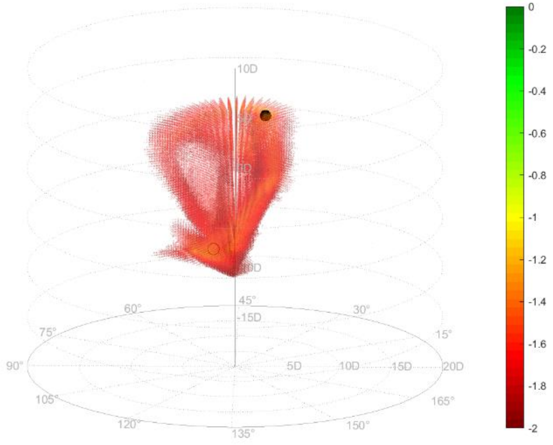

SyntEye KTC 2 Scleral lens correction

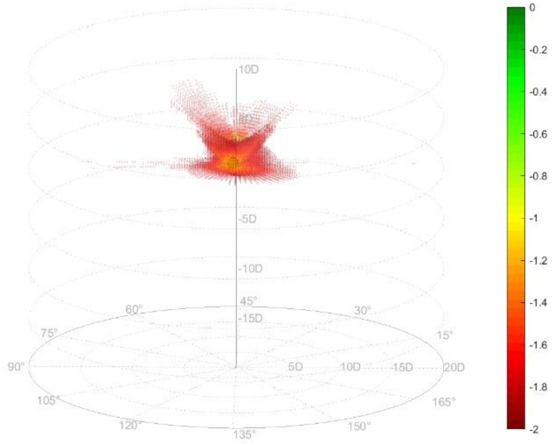

SyntEye KTC 3 Spectacle correction

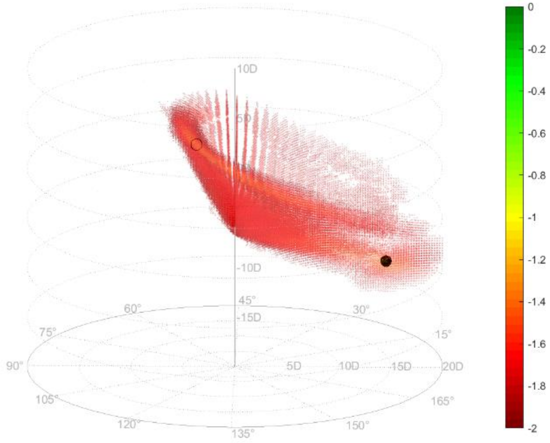

SyntEye KTC 3 Scleral lens correction

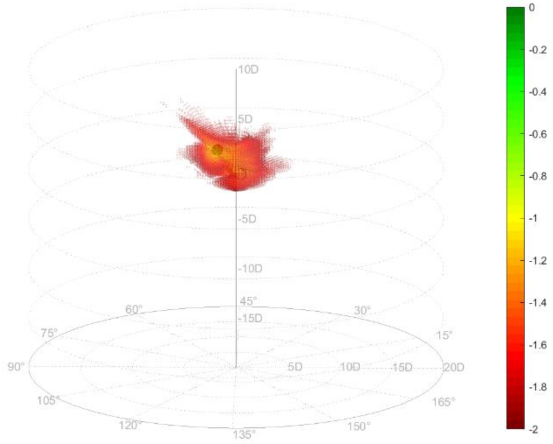

SyntEye KTC 4 Spectacle correction

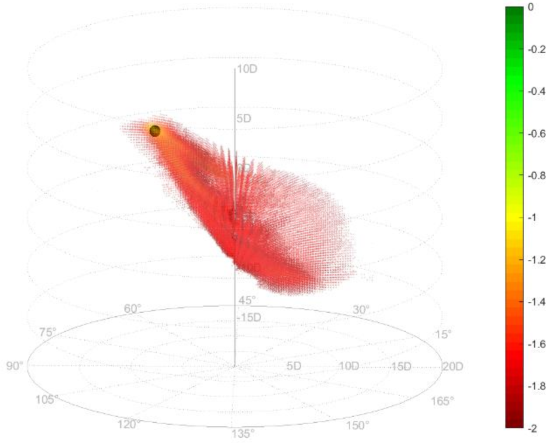

SyntEye KTC 4 Scleral lens correction

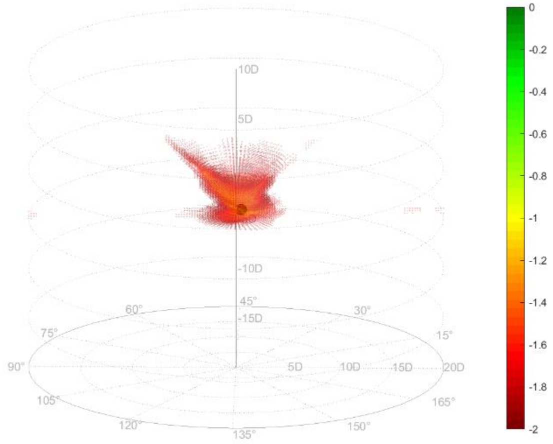

SyntEye KTC 6 Spectacle correction

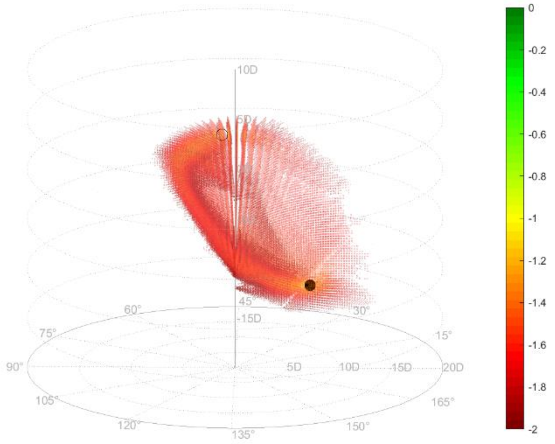

SyntEye KTC 6 Scleral lens correction

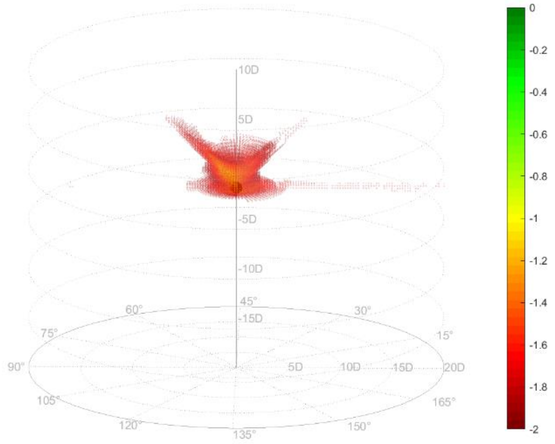

SyntEye KTC 7 Spectacle correction

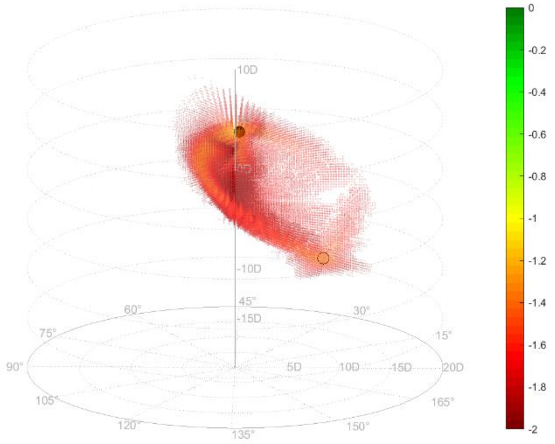

SyntEye KTC 7 Scleral lens correction

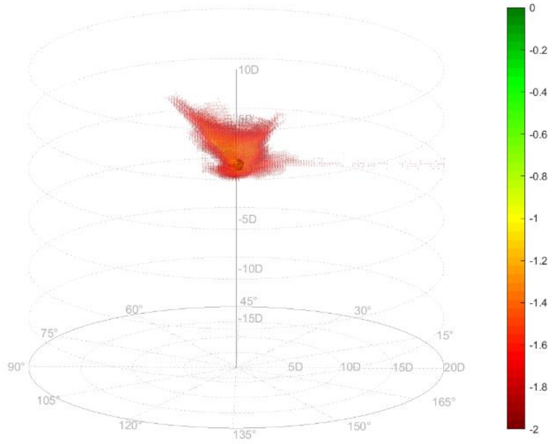

SyntEye KTC 9 Spectacle correction

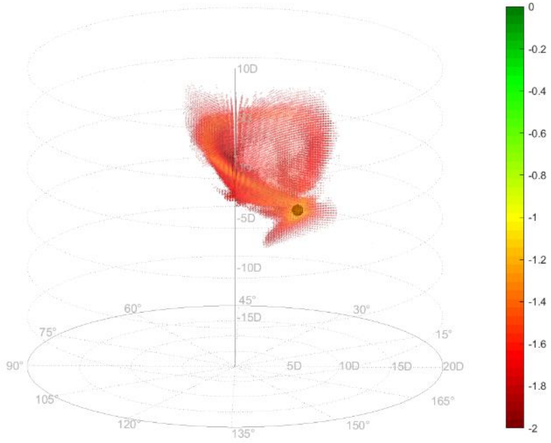

SyntEye KTC 9 Scleral lens correction

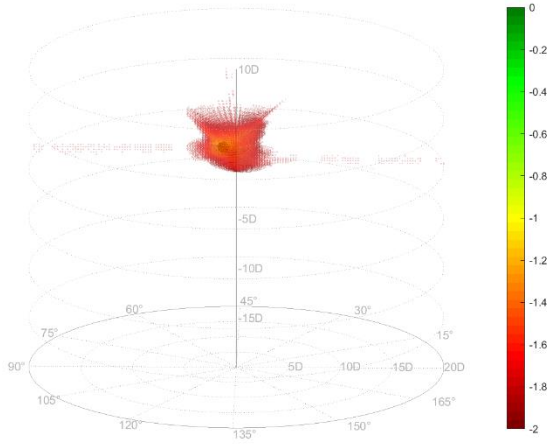

SyntEye KTC 10 Spectacle correction

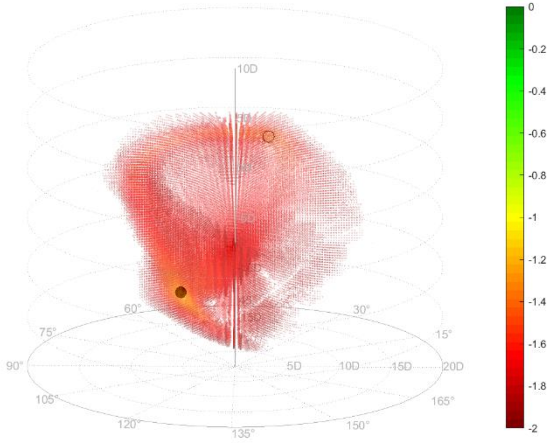

SyntEye KTC 10 Scleral lens correction

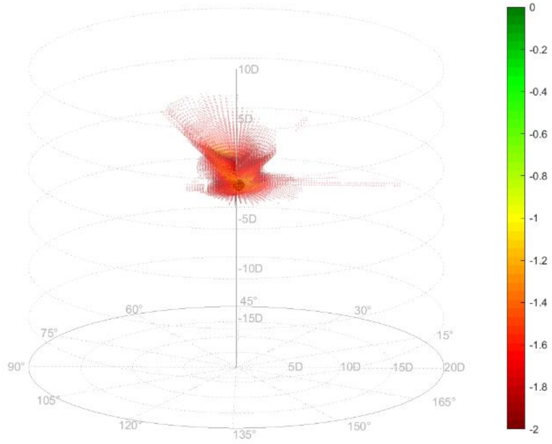

SyntEye KTC 14 Spectacle correction

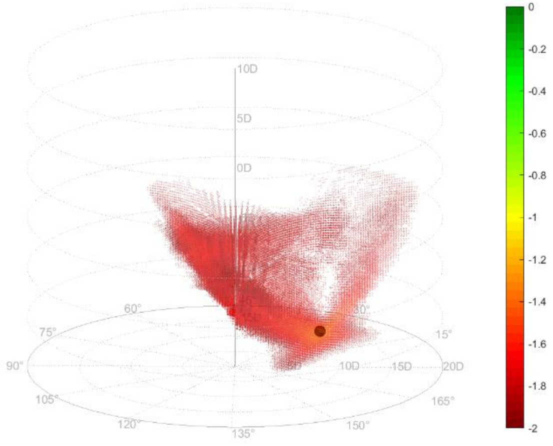

SyntEye KTC 14 Scleral lens correction

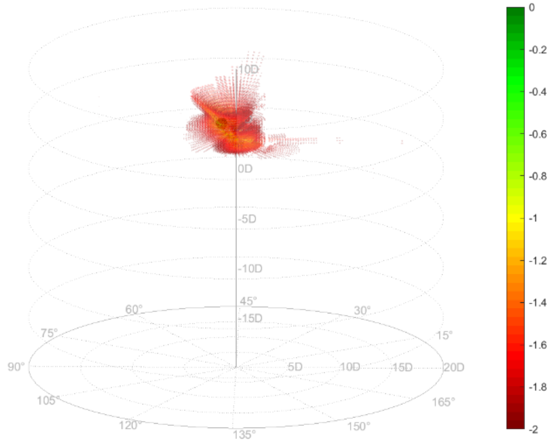

SyntEye KTC 15 Spectacle correction

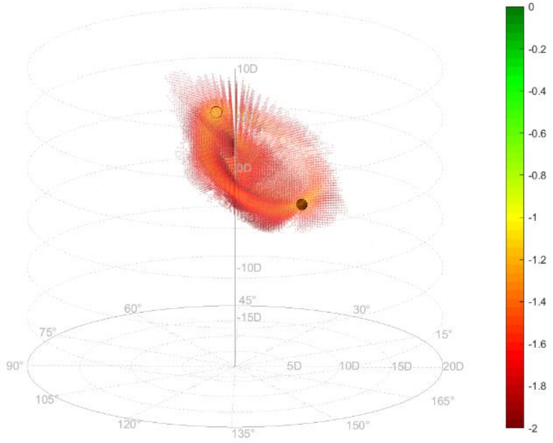

SyntEye KTC 15 Scleral lens correction

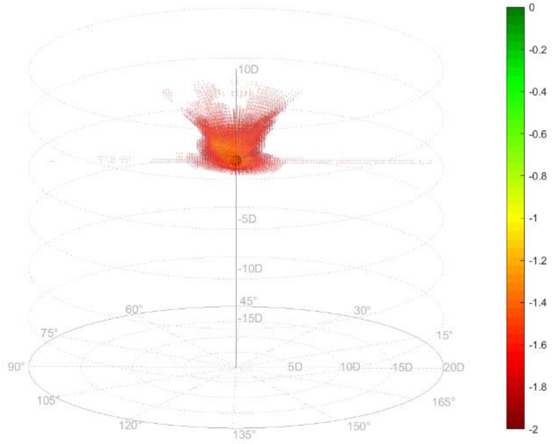

SyntEye KTC 16 Spectacle correction

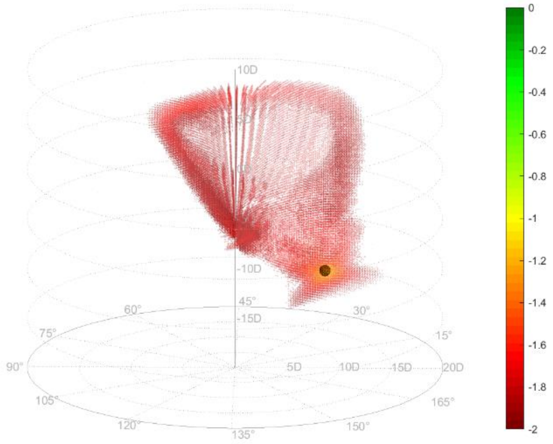

SyntEye KTC 16 Scleral lens correction

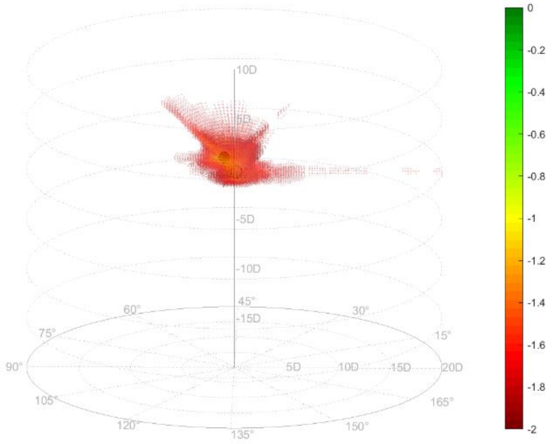

SyntEye KTC 17 Spectacle correction

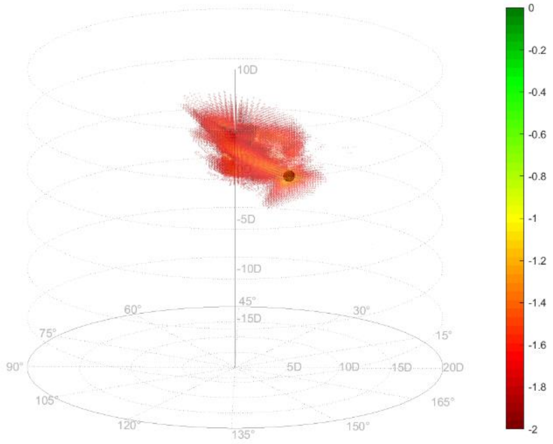

SyntEye KTC 17 Scleral lens correction

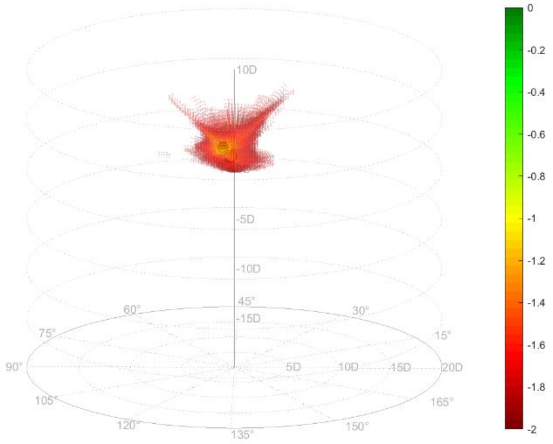

SyntEye KTC 18 Spectacle correction

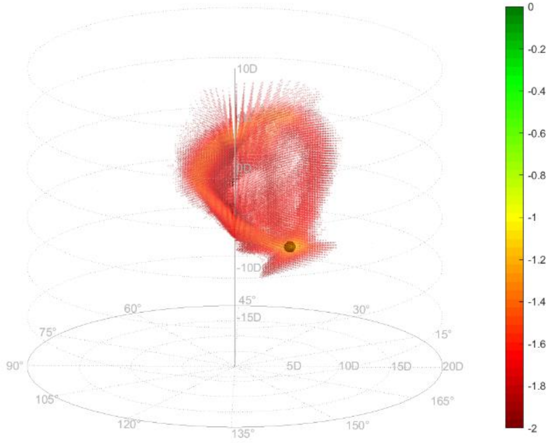

SyntEye KTC 18 Scleral lens correction

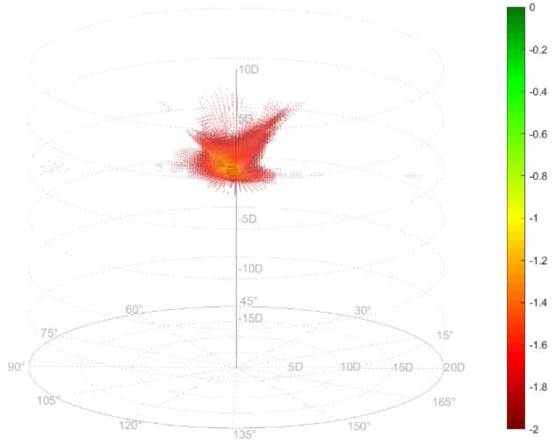

SyntEye KTC 21 Spectacle correction

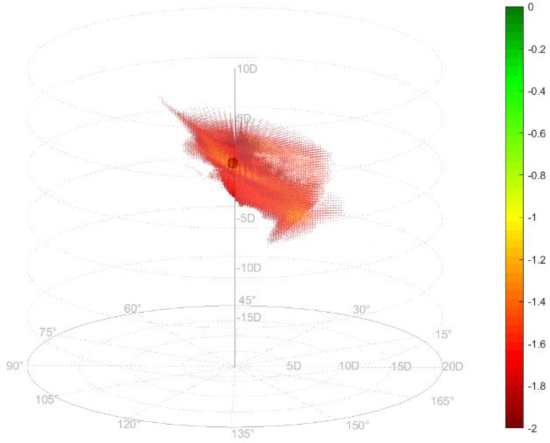

SyntEye KTC 21 Scleral lens correction

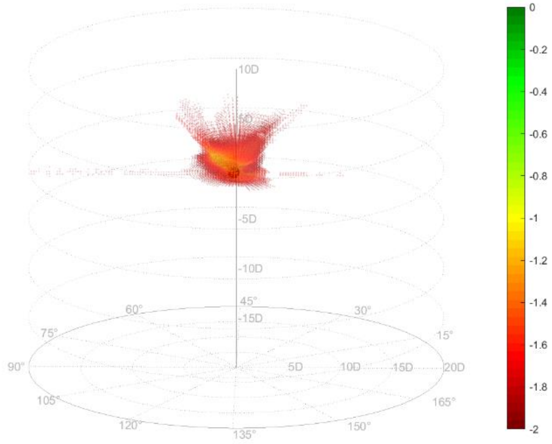

SyntEye KTC 22 Spectacle correction

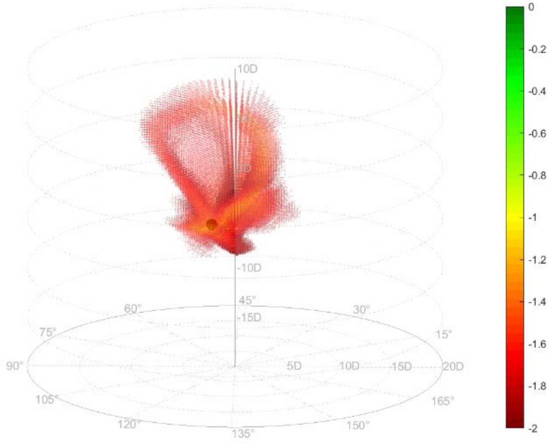

SyntEye KTC 22 Scleral lens correction

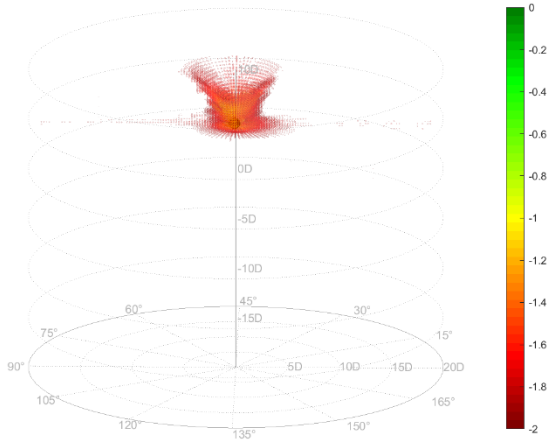

SyntEye KTC 23 Spectacle correction

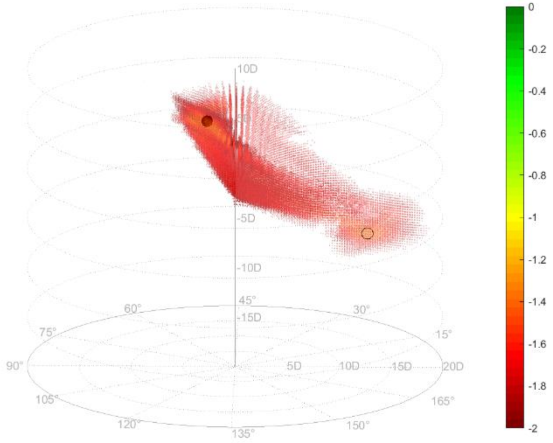

SyntEye KTC 23 Scleral lens correction

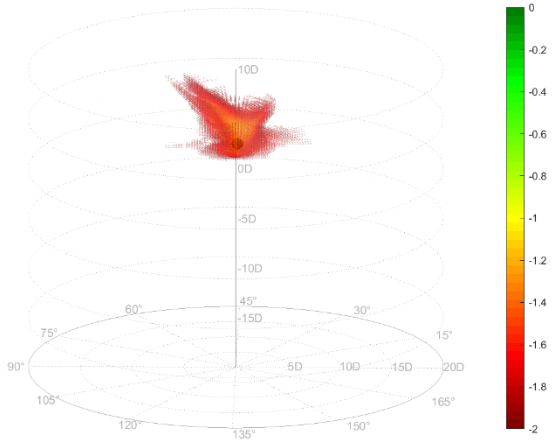

SyntEye KTC 24 Spectacle correction

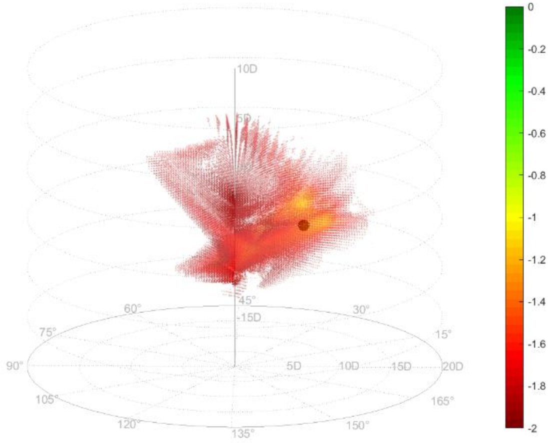

SyntEye KTC 24 Scleral lens correction

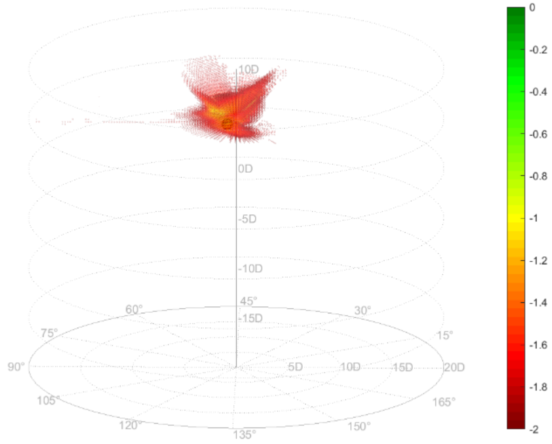

SyntEye KTC 26 Spectacle correction

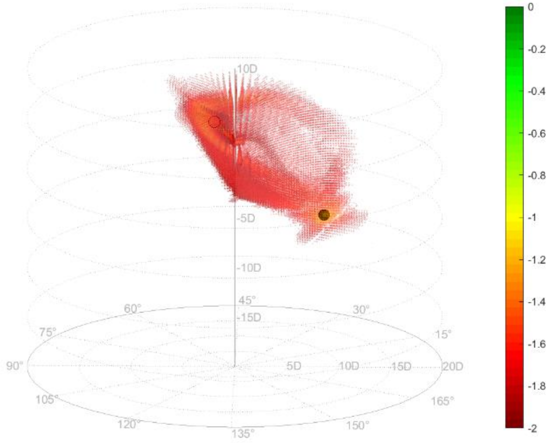

SyntEye KTC 26 Scleral lens correction

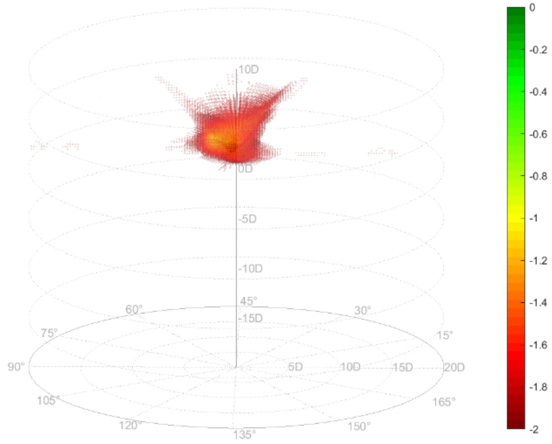

SyntEye KTC 27 Spectacle correction

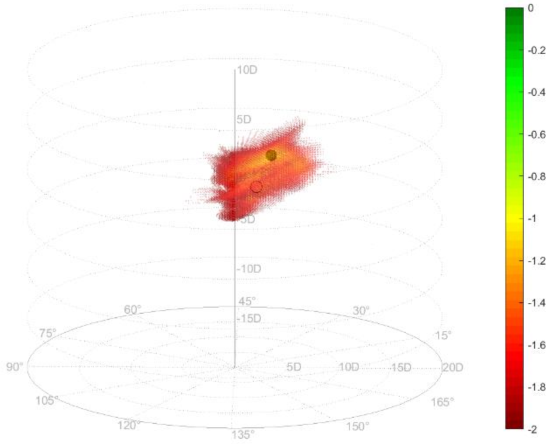

SyntEye KTC 27 Scleral lens correction

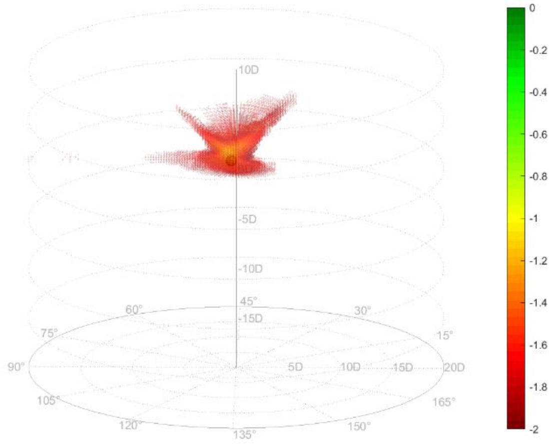

SyntEye KTC 28 Spectacle correction

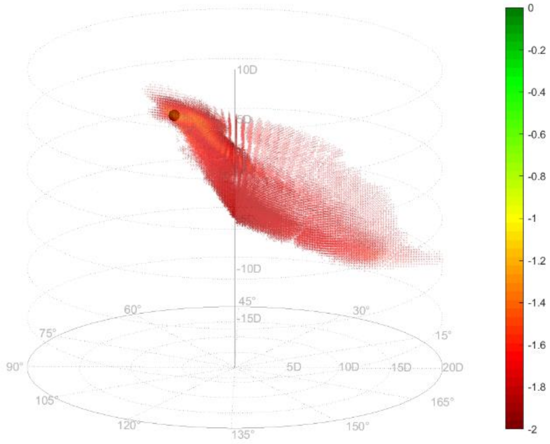

SyntEye KTC 28 Scleral lens correction

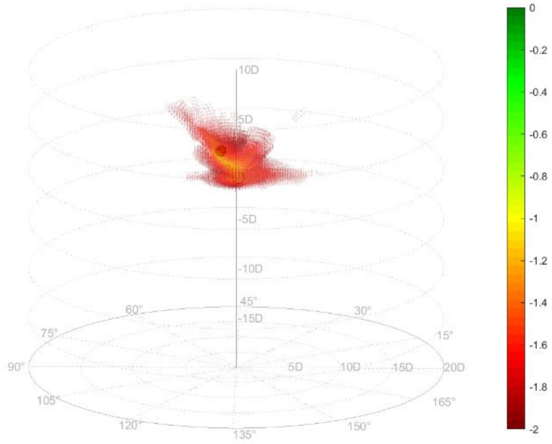

Supplement: Supplement 1 [file jovi-21-10-18_s001.pdf]
